# Discrepancy in efficiency scores due to sampling error in data envelopment analysis methodology: evidence from the banking sector

**DOI:** 10.12688/f1000research.153125.1

**Published:** 2024-08-06

**Authors:** Kishore L, Geetha E, Shivaprasad S P, Sachin R Chandra

**Affiliations:** 1 Department of Commerce, Manipal Academy of Higher Education, Manipal, Karnataka, 576104, India; 2 School of Business, RV University, Bengaluru, Karnataka, 560059, India

**Keywords:** Sampling Error; Performance Evaluation; Efficiency; Data Envelopment Analysis; Non-parametric Tests; Banking industry

## Abstract

**Background:**

Data Envelopment Analysis (DEA) methodology is considered the most suitable approach for relative performance efficiency calculation for banks as it is believed to be superior to traditional ratio-based analysis and other conventional performance evaluations. This study provides statistical evidence on the sampling error that can creep into performance evaluation studies using the DEA methodology. Inferences are drawn based on samples, and various preventive measures must be taken to eliminate or avoid sampling errors and misleading results. This study demonstrates the possibility of sampling error in DEA with the secondary data available in financial statements and reports from a sample set of banks.

**Methods:**

The samples included 15 public sectors and five leading private sector banks in India based on their market share, and the data for calculating efficiencies were retrieved from the published audited reports. The sample data was collected from 2014 to 2017 because the banking sector in India witnessed a series of mergers of public sector banks post-2017, and the data after that would be skewed and not comparable due to the demonetization policy implementation and merger process-related consolidation implemented by the Government of India. The efficiency measures thus computed are further analyzed using non-parametric statistical tests.

**Results:**

We found statistically significant discrepancies in the efficiency score calculations using DEA approach when specific outlier values. Evidence is provided on statistically significant differences in the efficiencies due to the inclusion and exclusion of particular samples in the DEA.

**Conclusion:**

The study offers a novel contribution along with statistical evidence on the possible sampling error that can creep into the performance evaluation of organizations while applying the DEA methodology.

## Introduction

Performance measurement and productivity improvement are vital for any organization as they help assess their performance and compare with benchmark organizations or set benchmarks to identify weak areas and opportunities for performance enhancement. ^1^ Among the various performance indicators, efficiency is crucial and frequently used as an assessment tool against comparative benchmarks. Banks operate in a highly competitive market, and efficiency evaluation helps identify areas for improvement and develop sustainable strategies for performance enhancement, leading to a better competitive position in the industry. Financial ratio-based evaluation is adopted widely in performance assessment, which can be misleading as the benchmarks are arbitrary. ^2^ This shortfall has increased the adoption of frontier performance evaluation methods across industries, including the banking sector. ^3^^,^^4^ One commonly used non-parametric technique is Data Envelopment Analysis (DEA) in the banking industry. DEA is opined as the aptest approach for relative performance efficiency calculation for banks as DEA methodology is considered superior to traditional ratio-based analysis embraced for conventional performance evaluation. ^4^ However, inferences drawn with DEA methodology based on samples can lead to possible sampling error. ^5^^,^^6^ This study demonstrates the impact of such outlier samples that can skew the efficiency computed using DEA methodology with the secondary data available in financial statements and reports from a sample set of banks. The study offers a novel contribution along with statistical evidence on the possible sampling error that can creep into the performance evaluation of organizations while applying the DEA methodology.

### Literature review

As per DEA methodology, the efficiency of any service process or system can be computed as a ratio of output to input. ^7^ Considering several inputs and outputs, the efficiency of each decision-making unit (DMU) can be written as

$$\text{Efficiency of unit}\;j=\frac{u_{1}y_{1j}+u_{2}y_{2j}{+}_{\ldots \ldots \ldots }}{v_{1}x_{1j}+v_{2}x_{2j}{+}_{\ldots \ldots \ldots }}$$ (1)

where u _1_ is the weight given to the output 1, v _1_ is the weight given to the input 1, x _1j_ is the amount of input from unit j, y _1j_ is the amount of output from unit j

The model for efficiency computation considering the multiple outputs and multiple inputs based on the above is given by

$$\max E_{m}=\frac{\sum_{j=1}^{J}v_{\mathrm{jm}}y_{\mathrm{jm}}}{\sum_{i=1}^{I}u_{\mathrm{im}}x_{\mathrm{im}}}$$ (2)

subject to

$$\begin{array}{c} 0\leq \frac{\sum_{j=1}^{J}v_{\mathrm{jm}}y_{\mathrm{jn}}}{\sum_{i=1}^{I}u_{\mathrm{im}}x_{\text{in}}}\leq 1;n=1,2,K,N\\ v_{\mathrm{jm}},u_{\mathrm{im}}\geq 0;i=1,2,K,I;j=1,2,K,J \end{array}$$

Where “E _m_ is the efficiency of the mth DMU”, “y _jm_ is the output of the mth DMU”, “v _jm_ is the weight of the output”, “x _im_ is the input of the mth DMU”, “u _im_ is the weight of the input” and “y _jn_ and x _in_ are the j ^th^ output and i ^th^ input”

Based on the Linear Programming problem technique, DEA computes efficiency with two assumptions of return to scale - constant and variable, ^7^ where constant return to the scale-based assumption of DEA is referred to as the overall technical efficiency. At the same time, variable returns to scale decompose overall technical efficiency into two components: pure technical efficiency and scale efficiency. ^8^

The available literature on the discrepancies that can happen in DEA methodology is very scant. Those discussions on variation in performance efficiency are primarily about sample size and the possible interpretation errors during decision-making. Zhang & Bartels ^8^ analyzed the variation in efficiency based on sample size using Monte Carlo Simulation. Using data from electricity distribution companies, they evaluated the efficiency and proved that the estimated mean technical efficiency decreases with increased sample size. The impact of sample size on the results of models involving non-discretionary variables was studied by Staat. ^8^ Smith ^5^ discussed the model misspecification in DEA, specifically when the sample size is insignificant. Banker and Chang ^8^ discussed the procedure to detect and remove the outliers in the DEA approach. However, their approach was to remove selected observations or samples. However, the variation that can creep in due to the inclusion and exclusion of a specific set of samples is not considered. While Johnson & McGinnis ^6^ focused on the mathematical model of inefficient outlier detection, Chen & Johnson ^9^ developed a mathematical model to detect efficient and inefficient outliers in efficiency computation.

There is a significant amount of research on the performance evaluation of banks based on DEA. ^2^^–^^4^^,^^10^^–^^19^ These prior studies have given insight into the efficiency of the performance of private and public sector banks in different contexts. These studies using DEA methodology have computed the efficiency based on the sample data collected from financial reports and published reports, and inferences are made according to the efficiency output from DEA methodology. However, the sampling error that can creep into performance evaluation studies using DEA methodology needs to be addressed, as it often gives misleading results regarding the efficiency of sample organizations under study. More precisely, which decision-making units under study must be chosen as the sample in the survey? The DEA methodology is a relative efficiency evaluation technique, and it will lead to misleading extrapolation when proper samples are not considered. Therefore, the problem statement framed is “Do sampling errors result in misleading results in performance evaluation using DEA methodology?

The study aims to critically analyze any significant difference in the efficiency scores due to the inclusion or exclusion of specific sample(s) in DEA methodology-based studies. State Bank of India (SBI) is the outlier to test the hypothesis, as stated below.

Ho: “ *There is no significant difference in the average efficiency scores between the two groups, SBI included and excluded, respectively.”*

Ha: “ *There is a significant difference in the average efficiency scores between the two groups, SBI included and excluded, respectively.”*

## Methods

The performance efficiency scores of banks are calculated using DEA methodology. ^20^ The study sample comprises 20 selected banks in India, which include fifteen public sector and five leading private sector banks based on their market share. The data for analysis for all 20 banks were retrieved from the published audited reports and Profit & Loss statements of banks available on the respective bank websites. ^21^ The period of data collected is from 2014 to 2017 for all the 20 banks selected, which is before the demonetization policy implementation by the Government of India ^22^ and before the mergers of many public sector banks in India ^23^ so that the effects of such a major national-level policy implementation and mergers do not have any impact on the data analysis. Overall technical efficiency based on DEA methodology was computed with the input variables as total and employee expenses and net Non-Performing-Assets (NPAs), considering the two critical variables per the literature review: total income and operating profit as outputs. Efficiency was computed in two groups, with one group of DMUs including SBI (State Bank of India) and another group excluding SBI. SBI is selected for comparison sample as the values of all the variables for SBI considered in the study indicate it as an outlier and, hence, the most suitable sample to compare. The results were analyzed to study the efficiency distribution after the computation of performance efficiency scores of the DMUs. Further, the Mann-Whitney U test is adopted to test the differences in the average distribution between the two groups. ^24^ The free version of the DEAFrontier tool ^24^ was used to calculate efficiency scores using DEA methodology, and the open-source R programming software was used to perform the non-parametric hypothesis test. Data visualization was performed using Python open-source programming software.

## Results and Discussion

**Figure 1.**
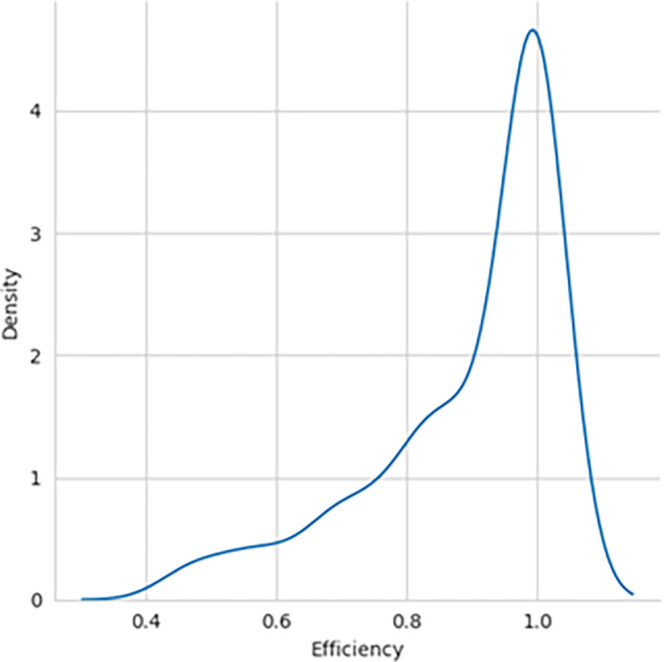
Performance efficiency distribution - SBI included.

**Figure 2.**
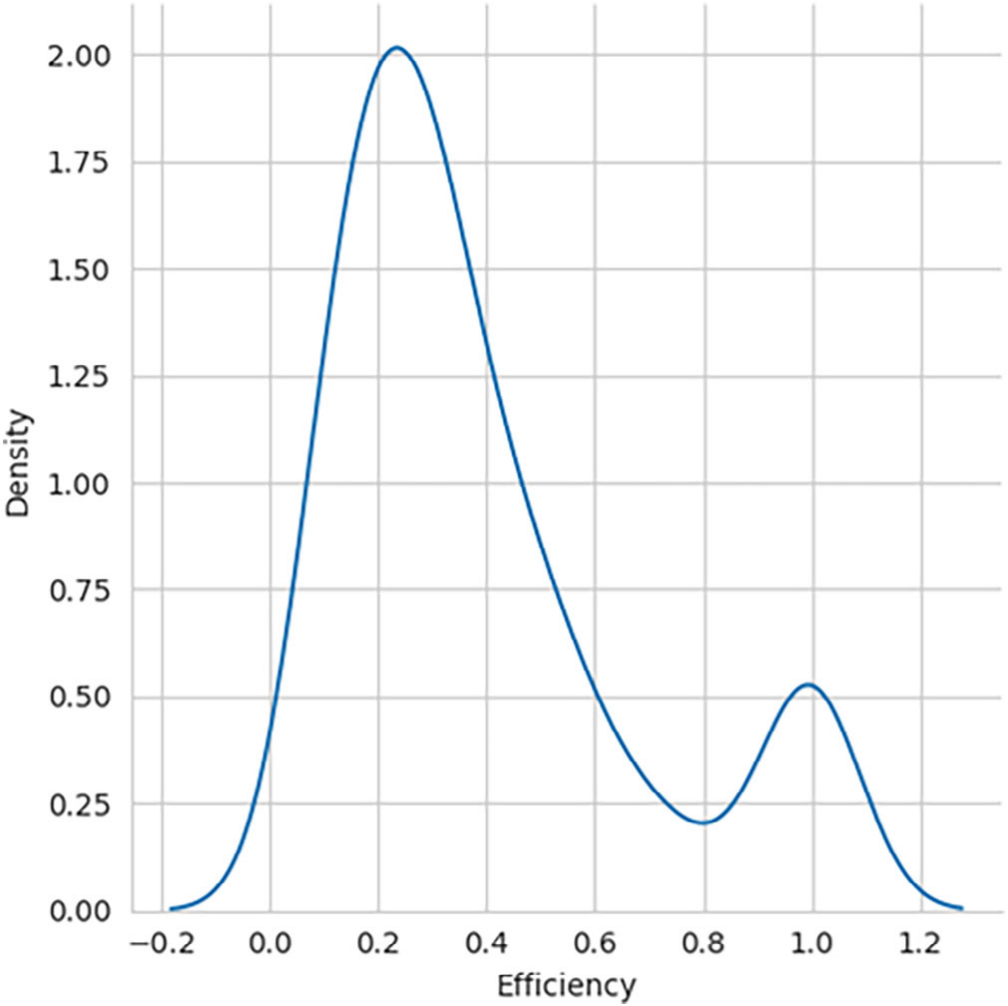
Performance efficiency distribution - SBI excluded.

**Figure 3.**
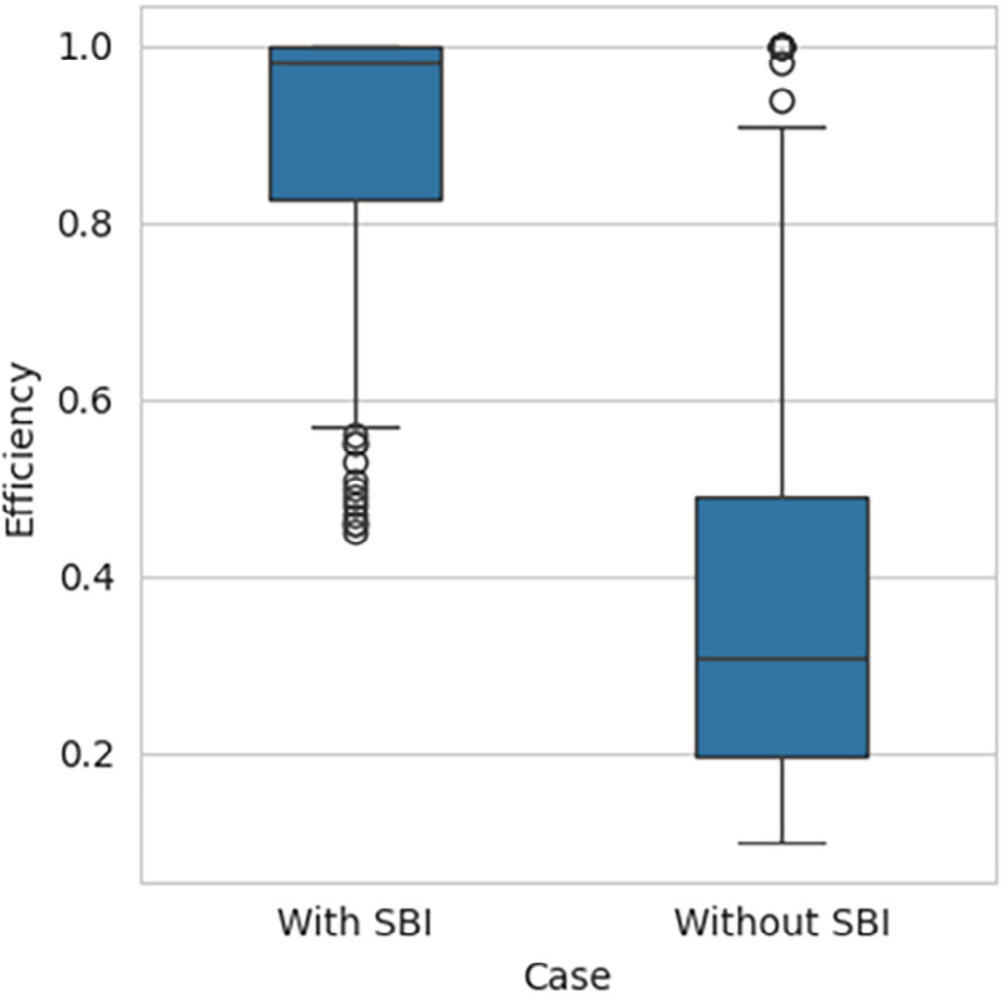
Box Plot for SBI included and excluded.

The performance efficiency score was calculated using DEA methodology, with the SBI sample included and later excluded for comparative analysis. The histogram showing the performance efficiency of the samples – with SBI included and SBI excluded, as shown in Figure 1 and Figure 2, respectively, indicates that the efficiency scores are not normally distributed. The box plot for the performance efficiency scores, with SBI included and SBI excluded, shown in Figure 3, also confirms that the efficiency scores are not normally distributed.

Shapiro-Wilk Test is performed to check for normal data distribution, and the test results are shown in Table 1. W-static and p-value for both SBI included and excluded confirm that the distributions are not typical. Skewness and Kurtosis measures also indicate that the efficiency of samples with SBI included having negatively skewed distribution and without SBI included as positively skewed.

**Table 1.** Shapiro-Wilk Normality Test result.

Measure	SBI included	SBI excluded
W	0.77683	0.82856
p-value	2.2e ^−16^	3.8392e ^−15^
Skewness	-1.29	1.19
Kurtosis	0.736	0..325

Since the data is abnormal, non-parametric tests are applied to test the hypothesis. The results of the Mann-Whitney U test for independent samples test are shown in Table 2. The p-value is almost equal to 0 at a 0.05 significance level.

**Table 2.** Mann-Whitney test for independent samples Test result.

	Minimum	25%	Median	75%	Maximum	p-value
SBI included	0.4492	0.8273	0.9800	1.000	1	5.34e ^−52^
SBI excluded	0.0954	0.1986	0.3068	0.4923	1	

Interpreting from Table 2, we can reject Ho, indicating that there is a statistically significant difference in efficiency distribution between the performance efficiency calculated with SBI included and excluded. Wilcoxon signed-rank test with continuity correction ^25^^,^^26^ was also performed to test the following hypothesis:

H _0_:
*“There is no significant difference in the efficiency score calculated with SBI included and excluded.”*

H _a_:
*“There is a significant difference in the efficiency score calculated with SBI included and excluded.”*

The p-value is almost 0 and is <0.05. Therefore, we fail to accept Ho, indicating a statistically significant difference in efficiency values computed with SBI and without SBI being considered in the sample. Spearman’s rank and Kendall Tau correlation tests are also conducted with the following hypotheses to increase the power of a statistical test.

H _0_:
*“There is no association between the efficiency values”*

H _a_:
*“There is a significant association between the efficiency values”*

**Figure 4.**
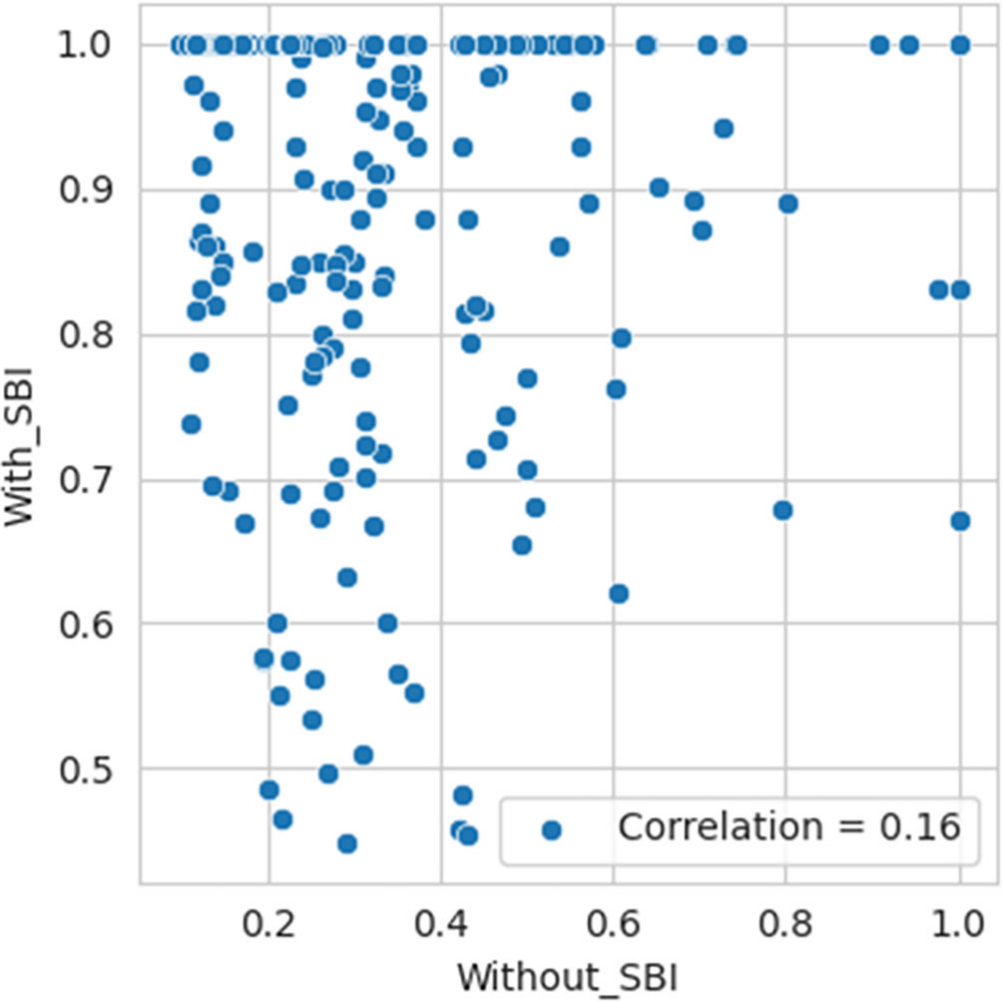
The correlation plot of SBI included and excluded.

**Figure 5.**
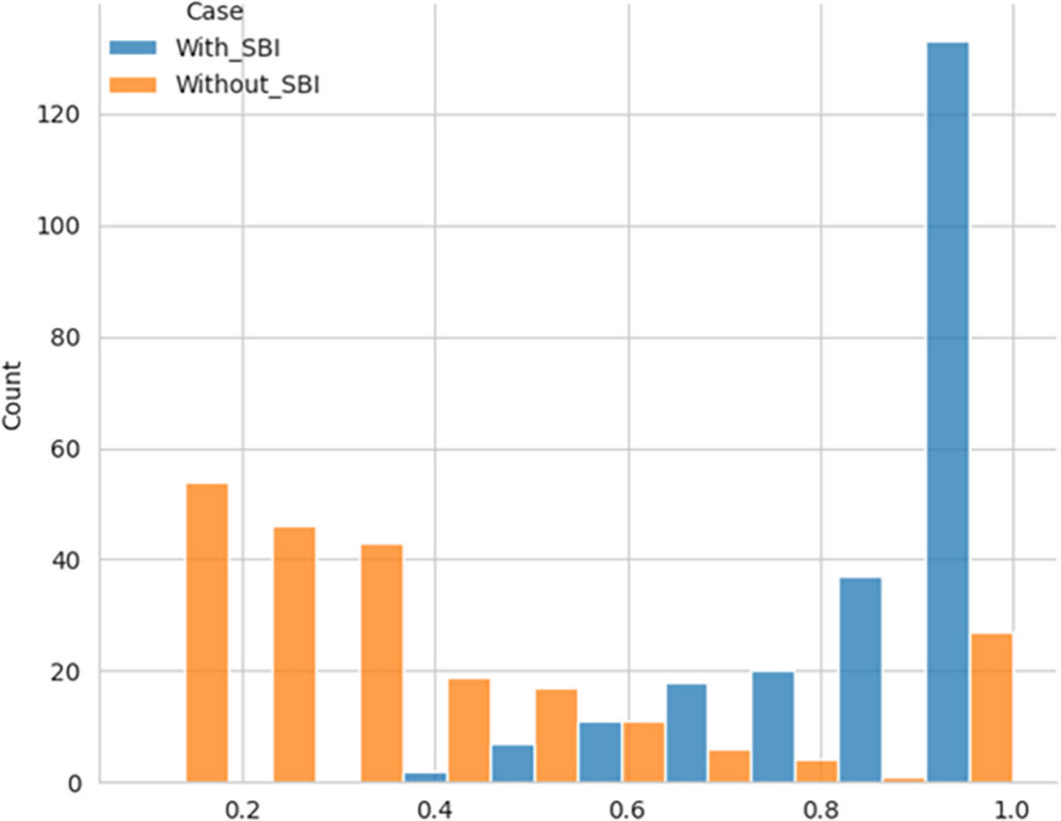
Bar diagram of SBI included and excluded.

According to Spearman’s rank correlation test, the p-value is almost 0.3671 and >0.05. Also, *rho* is very negligible, 0.06. Therefore, we fail to reject Ho, indicating no statistically significant association between the efficiency values computed with SBI and those without SBI being considered in the sample. Per the Kendall Tau correlation test, the p-value is almost 0.3165 and >0.05. Also, tau is very negligible, 0.048. Therefore, we fail to reject Ho, indicating no statistically significant association between the efficiency values computed with SBI and those without SBI being considered in the sample. The correlation plot and bar diagram shown in Figure 4 and Figure 5, respectively, also confirm the absence of any association between the efficiency values computed with SBI and those without SBI being considered in the sample.

**Figure 6.**
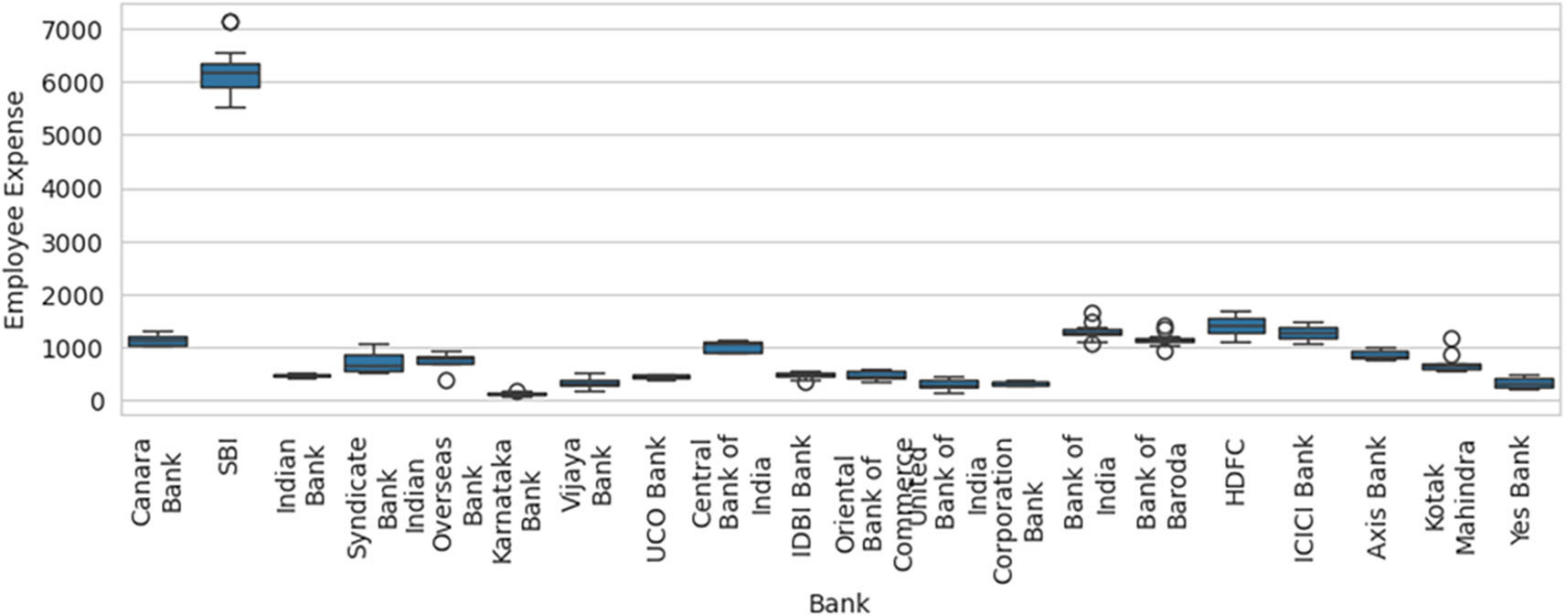
Box-and-whisker plot with SBI included.

**Figure 7.**
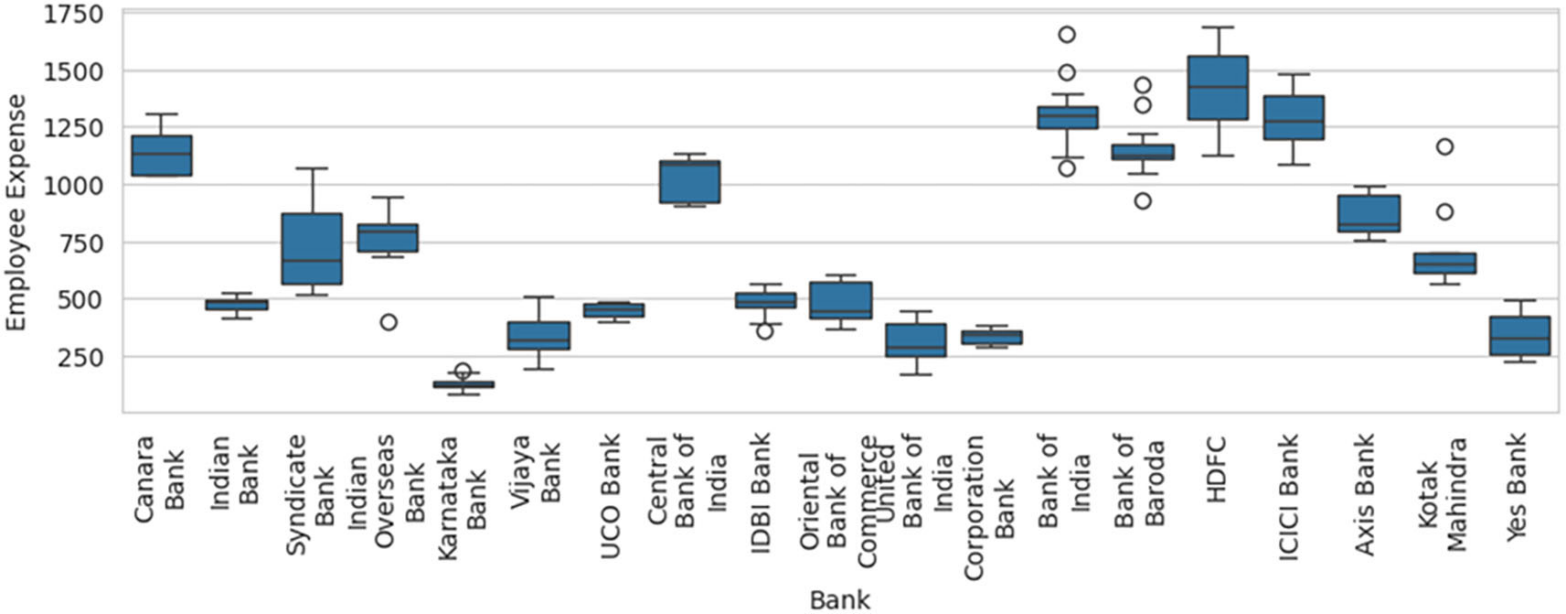
Box-and-whisker plot with SBI excluded.

Therefore, inclusion and exclusion of a sample result in significant variation in the efficiency computed, which in statistical terms is sampling error. Blind consideration of sample units for performance efficiency computation will result in misleading results, flawed interpretations, and erroneous decisions. Before calculation of efficiency using DEA methodology, the box-and-whisker plot for one of the variable Employee Expenses with the outlier SBI included, as shown in Figure 6, along with basic descriptive statistical measures, can help to identify the outliers that can result in efficiency computation errors due to sampling. The box-and-whisker with SBI excluded, as shown in Figure 7, shows how the variables are distributed in a range that allows comparison and efficiency evaluation to be more realistic.

## Conclusion

The relative efficiencies were calculated in two groups; one group included SBI as a DMU, and the other excluded SBI. The efficiencies thus calculated were tested for significant change in the average efficiency between the groups. The study provides statistical evidence on the sampling error that can creep into performance evaluation studies' DEA methodology. In practice, inferences were made based on samples, and various preventive measures must be taken to eliminate or avoid sampling error and related misleading results. The study offers a novel contribution along with statistical evidence on the possible sampling error that can creep into the performance evaluation of organizations due to the inclusion or exclusion of specific samples in the relative performance evaluation computation using DEA. The study also discussed how box-and-whisker can help quickly to identify the possible outlier that can cause skewed efficiency scores due to sampling error. The study was limited only to major and selected sample banks. Future studies can consider all types of banks to get a holistic picture of the banking industry performance benchmark and outlier impacts. Future studies can adopt other relevant non-parametric tests and thus increase the statistical power to confirm the findings.

### Ethical approval and consent

Ethical approval and consent were not required.

## Data Availability

The underlying data of the variables used for performance efficiency score calculation is taken from the audited reports of banks published on the respective bank websites and accessible to the public since the sample companies are registered and listed. For example, the data of SBI Bank is available in the PDF format reports published in the link https://sbi.co.in/web/investor-relations/reports. The user can navigate and select the appropriate year to download the reports in PDF format and retrieve data as required. The links for accessing other sample banks are given below. https://www.canarabank.com/pages/audited-and-unaudited-financial-results https://www.iob.in/Financial_perf https://karnatakabank.com/investors/quarterly-results https://ucobank.com/financial-results-2014-15 https://centralbankofindia.co.in/en/node/219217 https://www.idbibank.in/idbi-bank-analyst-presentation.aspx https://www.unionbankofindia.co.in/english/financial-result.aspx https://bankofindia.co.in/financial-result https://www.bankofbaroda.in/shareholders-corner/financial-reports/financial-report-q4-2022-23 https://www.hdfcbank.com/personal/about-us/investor-relations/financial-results https://www.icicibank.com/about-us/qfr https://www.axisbank.com/shareholders-corner/financial-results-and-other-information/quarterly-results https://www.kotak.com/en/investor-relations/financial-results/quarterly-results.html?source=website https://www.yesbank.in/about-us/investor-relations/financial-information/financial-results For easy access, the data collected from the reports of all the sample banks considered for the study are consolidated and made available in the Figshare repository per the details below. Figshare: Audited reports data, https://doi.org/10.6084/m9.figshare.26046277. ^27^ This project contains the following data:
• Audited reports data in xlsx file format containing the data required for analysis taken from the banks website. Audited reports data” file contains all the data taken from audited reports that are required for analysis. • Quarterly performance efficiency scores calculated based on the data from audited reports. “Quarterly efficiency score” file contains the calculated performance efficiency scores using DEAFrontier software. Data are available under the terms of the Creative Commons Attribution 4.0 International license (CC-BY 4.0).

## Software availability

The free version DEAFrontier tool, available at https://www.deafrontier.net/deafree.html, was used to calculate performance efficiency scores using DEA methodology. The open-source R programming software, available at https://cran.r-project.org/bin/windows/base/, was used for the non-parametric hypothesis test.
