# Supplementary material for: Discrepancy in efficiency scores due to sampling error in data envelopment analysis methodology: evidence from the banking sector
Source: F1000Res. 2024 Nov 25;13:890. Originally published 2024 Aug 6. [Version 2] doi: 10.12688/f1000research.153125.2 (PMC11829147; doi:10.12688/f1000research.153125.2)
Supplement: Peer review history

# Reviewer report: Reviewer response for version 2

**DOI:** 10.5256/f1000research.174428.r360607

**Published:** 2025-02-14

**Authors:** Yossi Hadad

**Affiliations:** 1 Sami Shamoon College of Engineering, Beer-Shvea, Israel

The study addresses performance evaluation using DEA methodology (CCR model): The impact of sampling errors. As highlighted in the article, the focus on statistical evidence and examination of outliers fills a critical gap in the literature. Statistical methods, including the Mann-Whitney U test, Wilcoxon signed rank test, and correlation analyses, are used, adding credibility to the findings. The inclusion of private and public sector banks from India ensures a reasonable sample diversity. At the same time, the period before significant policy changes (e.g., demonetization and mergers) minimizes extraneous noise in the data. Practical contribution: Identifying outliers using visual tools such as box and whisker plots is a practical contribution that practitioners can quickly implement. A discussion of the implications for decision-makers and policymakers highlights the real-world applicability of the study. The article provides new insights into how the inclusion or exclusion of specific decision-making units (DMUs) can bias efficiency calculations.

**Comments and Suggestions for Authors:**

 1) The paper requires rewriting and editing. The contribution of the manuscript was not clear. A strong competitive analysis is needed. What are the contributions to research by writing your manuscript?  Make the exposition tight and clear (right now, it is meandering and redundant, particularly in the first part of the paper).  The background is not well organized. It would be better if the authors first explained the motivation for their study, then discussed the problem statement, and finally reviewed the relevant studies.

 2)  In the conclusions section, the authors should provide a general interpretation of the rustles, the unique contributions of the paper, limitations of the research, managerial implications, and the impact that the paper might have on future research and on policy decisions.

 3) Page 1- Change equations 1) and 2) to the following formulation:











        

**4) It is important to note that:  **Model (1) - The CCR model (Charnes et al., 1978) assumes that the production function exhibits constant returns-to-scale (RTS). The BCC developed by Banker, Charnes, and Cooper (BCC model) adds an additional constant variable, , in order to permit variable returns-to-scale.

 5) An explanation should be provided as to why you used the CCR model rather than the BCC model.

 6) The authors should add the following paragraph: “The study focuses exclusively on Indian banks, which limits the generalizability of findings to other countries or industries with different operational contexts (limited generalizability). Although the study examines key input and output variables (e.g., total revenue, operating profit, total expenses), it is possible that adding variables such as customer satisfaction or adoption of digital banking could provide a more holistic assessment of efficiency (narrow scope of analysis). This is simplistic and mainly involves identifying outliers using descriptive statistics and visualization. It is possible that using a BCC model rather than a CCR model, adding constraints on the weights (a non-Archimedean quantity) could provide deeper insights into the impact of outliers. Although non-parametric tests are appropriate given the distribution of the data, additional statistical techniques could have been applied to strengthen the findings. A broader set of banks in multiple countries or regions can increase the external validity of the findings. Combining advanced DEA techniques: using methods such as bootstrapped DEA or stochastic DEA (see, for example, Olesen & Petersen, 2016). or ranking methods using DEA (see, for example, Hadad, &. Hanani,2011).  Including dynamic variables (e.g., year-over-year growth) and non-financial factors (e.g., environmental and social governance indicators) can add depth to the efficiency analysis."

**Add to the References:**

 Olesen, O. B., & Petersen, N. C. (2016 [Ref-1]). Stochastic data envelopment analysis—A review. *European journal of operational research*, *251*(1), 2-21.

 ‏

 Hadad, Y., & Hanani, M. Z. (2011 [Ref-2]). Combining the AHP and DEA methodologies for selecting the best alternative. *International journal of logistics systems and management*, *9*(3), 251-267.

 ‏

 Nguyen, H. O., Nguyen, H. V., Chang, Y. T., Chin, A. T., & Tongzon, J. (2016 [Ref-3]). Measuring port efficiency using bootstrapped DEA: The case of Vietnamese ports, Maritime Policy and Management. 43(5), 644–659.)

Is the work clearly and accurately presented and does it cite the current literature?

Partly

If applicable, is the statistical analysis and its interpretation appropriate?

Yes

Are all the source data underlying the results available to ensure full reproducibility?

Yes

Is the study design appropriate and is the work technically sound?

Partly

Are the conclusions drawn adequately supported by the results?

No

Are sufficient details of methods and analysis provided to allow replication by others?

Yes

Reviewer Expertise:

Data Envelopment Analysis (DEA), work-study, ranking methods

I confirm that I have read this submission and believe that I have an appropriate level of expertise to confirm that it is of an acceptable scientific standard, however I have significant reservations, as outlined above.

## References

[1] : Stochastic Data Envelopment Analysis—A review. *European Journal of Operational Research* .2016;251(1) : 10.1016/j.ejor.2015.07.058 2-21 10.1016/j.ejor.2015.07.058

[2] : Combining the AHP and DEA methodologies for selecting the best alternative. *International Journal of Logistics Systems and Management* .2011;9(3) : 10.1504/IJLSM.2011.041687 10.1504/IJLSM.2011.041687

[3] : Measuring port efficiency using bootstrapped DEA: the case of Vietnamese ports. *Maritime Policy & Management* .2016;43(5) : 10.1080/03088839.2015.1107922 644-659 10.1080/03088839.2015.1107922

# Reviewer report: Reviewer response for version 1

**DOI:** 10.5256/f1000research.167974.r325801

**Published:** 2024-10-09

**Authors:** Weng Hoe Lam

**Affiliations:** 1 Universiti Tunku Abdul Rahman, Kampar, Malaysia

The study offers a novel contribution along with statistical evidence on the possible sampling error that can creep into the performance evaluation of organizations while applying the DEA methodology. Kindly refer to the following comments for improvement.

 1)Please include the managerial and policy implications section

 2)Please include the state-of-the-art table in the literature review section to show the research gap

Is the work clearly and accurately presented and does it cite the current literature?

Yes

If applicable, is the statistical analysis and its interpretation appropriate?

Yes

Are all the source data underlying the results available to ensure full reproducibility?

Yes

Is the study design appropriate and is the work technically sound?

Yes

Are the conclusions drawn adequately supported by the results?

Yes

Are sufficient details of methods and analysis provided to allow replication by others?

Yes

Reviewer Expertise:

Statistical modeling, Operations Research

I confirm that I have read this submission and believe that I have an appropriate level of expertise to confirm that it is of an acceptable scientific standard.

# Response

**Published:** 2024-10-09

**Authors:** Kishore L

**Affiliations:** Department of Commerce, Manipal Academy of Higher Education, Manipal, Karnataka, India

Dear reviewer,

 Thanks for the review and comments.

 We will include implications in addition to tabulating the literature review highlighting the gap in the revision.

 Thanking once again.

 Regards

 Kishore L

# Reviewer report: Reviewer response for version 1

**DOI:** 10.5256/f1000research.167974.r325798

**Published:** 2024-09-30

**Authors:** Katerina Fotova Čiković

**Affiliations:** 1 University North, Koprivnica, Croatia

The topic is well formulated, relevant to the  area of interest and related to an important and current subject on global level.

 The abstract is well worded and provides a short, clear and effective introduction to the paper.

 Methodology is extensively introduced and each step of the process is presented. There are too many figures. I wonder if they are all beneficial?

 However, there is a need for sample clarification. How did you come up with this sample? What was the selection process of the banks entering the sample? The analyzed period is somewhat questionable (it is a period almost 7 years ago, and it may not be relevant anymore). 

 Do not use "we", "our" in the text. Instead, use 3rd person singular.

Is the work clearly and accurately presented and does it cite the current literature?

Yes

If applicable, is the statistical analysis and its interpretation appropriate?

Yes

Are all the source data underlying the results available to ensure full reproducibility?

Partly

Is the study design appropriate and is the work technically sound?

Yes

Are the conclusions drawn adequately supported by the results?

Yes

Are sufficient details of methods and analysis provided to allow replication by others?

Yes

Reviewer Expertise:

Finance, banking sector, M&A, DEA

I confirm that I have read this submission and believe that I have an appropriate level of expertise to confirm that it is of an acceptable scientific standard, however I have significant reservations, as outlined above.

# Response

**Published:** 2024-09-30

**Authors:** Kishore L

**Affiliations:** Department of Commerce, Manipal Academy of Higher Education, Manipal, Karnataka, India

**How did you come up with this sample? What was the selection process of the banks entering the sample?**

**Response to the above 2 query:**

 The study sample comprises 20 selected banks in India, which include fifteen public sector and five leading private sector banks. This was done to have representation of public as well as private sector banks in the same. And therefore, as mentioned in the methodology section which I quote " The study sample comprises 20 selected banks in India, which include fifteen public sector and five leading private sector banks based on their market share", the selection was based on their **market share. **Hope the explanation is satisfactory.

**The analyzed period is somewhat questionable (it is a period almost 7 years ago, and it may not be relevant anymore).**

**Response to the above query:**

 The period of data collected is from 2014 to 2017 for all the 20 banks selected. This period was selected because there was two national-level major policy implemented in India - one on Demonetization and another was the merger of many banks. Due to demonetization and mergers there was an overhaul in the banks and also after the mergers the balance sheet is consolidated due to which the data after these policy implementations will not be suitable to empirically prove our hypothesis. And therefore, as mentioned in the methodology section which I quote "The period of data collected is from 2014 to 2017 for all the 20 banks selected, which is before the demonetization policy implementation by the Government of India [23] and before the mergers of many public sector banks in India [24] so that the effects of such a major national-level policy implementation and mergers do not have any impact on the data analysis", we decided to use the data before these major reforms. Hope the explanation is satisfactory.

**Do not use "we", "our" in the text. Instead, use 3rd person singular.**

**Response to the above suggestion:**

 Completely agree to your suggestion. The correction will be incorporated in the revision. Thanks for the review.
